# Pediatric Trauma and Trauma Team Activation in a Swiss Pediatric Emergency Department: An Observational Cohort Study

**DOI:** 10.3390/children10081377

**Published:** 2023-08-11

**Authors:** Anouk Herren, Cameron S. Palmer, Markus A. Landolt, Markus Lehner, Thomas J. Neuhaus, Leopold Simma

**Affiliations:** 1 Department of Pediatrics, Children’s Hospital Lucerne, Spitalstrasse, CH-6000 Lucerne, Switzerland; 2 Department of Pediatrics, University’s Children Hospital Zurich, University of Zurich, Steinwiesstrasse 75, CH-8032 Zurich, Switzerland; 3 Trauma Service, The Royal Children’s Hospital, Melbourne, VIC 3052, Australia; 4 School of Public Health and Preventive Medicine, Monash University, Melbourne, VIC 3004, Australia; 5 Department of Psychosomatics and Psychiatry and Children’s Research Center, University Children’s Hospital Zurich, University of Zurich, Steinwiesstrasse 75, CH-8032 Zurich, Switzerland; 6 Division of Child and Adolescent Health Psychology, Department of Psychology, University of Zurich, Binzmuehlestrasse 14, CH-8050 Zurich, Switzerland; 7 Children’s Research Center, University Children’s Hospital of Zurich, University of Zurich, CH-8032 Zurich, Switzerland; 8 Department of Pediatric Surgery, Children’s Hospital Lucerne, Spitalstrasse, CH-6000 Lucerne, Switzerland; 9 Emergency Department, Children’s Hospital Lucerne, Spitalstrasse, CH-6000 Lucerne, Switzerland; 10 Emergency Department, University’s Children Hospital Zurich, University of Zurich, Steinwiesstrasse 75, CH-8032 Zurich, Switzerland

**Keywords:** emergency department, trauma, trauma team activation, major trauma, pediatric, child, adolescent, trauma center, pediatric emergency medicine

## Abstract

Background. Trauma is one of the most common causes of death in childhood, but data on severely injured Swiss children are absent from existing national registries. Our aim was to analyze trauma activations and the profiles of critically injured children at a tertiary, non-academic Swiss pediatric emergency department (PED). In the absence of a national pediatric trauma database, this information may help to guide the design of infrastructure, processes within organizations, training, and policies. Methods. A retrospective analysis of pediatric trauma patients in a prospective resuscitation database over a 2-year period. Critically injured trauma patients under the age of 16 years were included. Patients were described with established triage and injury severity scales. Statistical evaluation included logistic regression analysis. Results. A total of 82 patients matched one or more of the study inclusion criteria. The most frequent age group was 12–15 years, and 27% were female. Trauma team activation (TTA) occurred with 49 patients (59.8%). Falls were the most frequent mechanism of injury, both overall and for major trauma. Road-traffic-related injuries had the highest relative risk of major trauma. In the multivariate analysis, patients receiving medicalized transport were more likely to trigger a TTA, but there was no association between TTA and age, gender, or Injury Severity Score (ISS). Nineteen patients (23.2%) sustained major trauma with an ISS > 15. Injuries of Abbreviated Injury Scale severity 3 or greater were most frequent to the head, followed by abdomen, chest, and extremities. The overall mortality rate in the cohort was 2.4%. **Conclusions**: Major trauma presentations only comprise a small proportion of the total patient load in the PED, and trauma team activation does not correlate with injury severity. Low exposure to high-acuity patients highlights the importance of deliberate learning and simulation for all professionals in the PED. Our findings indicate that high priority should be given to training in the management of severely injured children in the PED. The leading major trauma mechanisms were preventable, which should prompt further efforts in injury prevention.

## 1. Background

Trauma ranks among of the most frequent causes of death in childhood [1]. According to the WHO, 830,000 children die from unintentional injuries every year [2]. In European countries, mortality rates from pediatric trauma are low, but involve around 13% of severely injured children [3]. Major trauma in childhood differs in many respects from adult trauma. Anatomic, physiological, and metabolic differences are well described [4]. Additionally, distinctive injury patterns are encountered because children engage in different activities from adults, and children’s activities differ widely during childhood [5].

Switzerland has no comprehensive dataset or registry for pediatric trauma. Little is known about local patterns of injury severity, frequency, or mechanisms of injury (MOI) [6]. Adult trauma care is provided in a decentralized fashion. Since 2011, adult major trauma care has been delivered in twelve designated centers across Switzerland; pediatric major trauma care is delivered in eight accredited, designated pediatric Level 1 centers [7]. Data entry in the Swiss Trauma Registry [8] is mandatory but excludes patients under 16 years. Some pediatric centers enter data into registries outside Switzerland that have no formal age restriction, such as the Trauma Audit and Research Network (TARN) in the UK and the Deutsche Gesellschaft fur Unfallchirurgie (DGU) registry in Germany [9].

Currently, information concerning epidemiology, incidence, and outcome of injured Swiss children are restricted to reports by the Federal Office of Statistics, insurance company data, and the non-governmental Swiss Council for Accident Prevention [9]. However, these publicly accessible datasets have inherent limitations for epidemiological and medical research, mainly due to lacking injury classification according to standardized international definitions.

To help close this knowledge gap, this paper analyzes severely injured children who present to a non-academic, tertiary pediatric emergency department (PED) that serves as a Swiss Level 1 pediatric trauma center. The primary endpoint of this analysis is to assess trauma activations and characterize the profiles of these severely injured patients, including their injury patterns, injury severity, imaging requirements, and the necessity for intensive care. The intention behind this investigation is to provide insights that could enhance training programs, preparedness efforts, infrastructure design, institutional processes, and policy development within healthcare institutions.

## 2. Methods

We performed a retrospective analysis of pediatric trauma patients in the local resuscitation database who presented to our pediatric emergency department (PED) between 1 January 2018 and 31 December 2019.

### 2.1. Setting

During the study period, tertiary, non-academic PED had an annual census of 21,000 patients. It is the only pediatric hospital in the geographically confined region of central Switzerland, and designated pediatric Level 1 trauma centers in Switzerland. Major trauma patients receive treatment in a trauma bay that is shared with the adult hospital on the same campus. This shared facility allows for immediate access to advanced imaging (CT and MRI) and interventional radiology.

The majority of Swiss pediatric hospitals utilize the five-level Australasian Triage Scale (ATS) as the standard tool for patient triage [10]. The PED is staffed with pediatricians trained in pediatric emergency medicine (PEM) from 08:00 to 23:00. Senior pediatric intensivists are available on site at all times. Senior pediatric surgeons are available during business hours and on call after hours (with a maximum response time of 30 min). The resuscitation team follows the principles of resuscitation algorithms of Advanced Trauma Life Support (ATLS^®^) and Pediatric Advanced Life Support (PALS^®^).

The team leader is an ATLS^®^-certified, senior doctor of the pediatric hospital. Depending on factors including level of experience, availability, and time of presentation, this role is taken by either PEM, pediatric surgery, or pediatric intensive care (PICU). The pediatric trauma team is composed of senior staff members from pediatric surgery, PED, PICU, and pediatric anesthesia.

The trauma team is activated through an internal phone conference with all staff required. These calls for trauma team activations (TTAs) are electronically logged. The institution’s trauma procedure adheres to ATLS^®^ principles and has been tailored based on prior publications [11].

### 2.2. Inclusion Criteria and Exclusion Criteria

We included all pediatric trauma presentations under 16 years of age that had been classified as critical. We used a hybrid definition for critical injury, in which patients were classified as critical if they met any one of five criteria:

- ATS category 1;
- admitted to PICU from PED, whether directly or via the operating room (OR);
- received TTA;
- transferred to an outside hospital;
- died in PED.

Exclusion criteria were trauma presentations not meeting the above criteria. Drownings without concern for mechanical trauma and trauma patients with non-trauma-related PICU admission such as complications during procedural sedation were removed from the sample.

### 2.3. Data Sources and Variables

The sources for data extraction were the separate ED information system, the electronic medical record (EMR), as well as the electronic TTA log. Data collected in the prospective resuscitation database included demographics and data relating to the injury event, ED reception, post-ED management, and in-hospital outcomes. Anatomical injuries were coded using the Abbreviated Injury Scale (AIS) Update 2008 (German version Trauma Register DGU^®^—V2015.1). Injury Severity Score (ISS) values were calculated and grouped into major and minor trauma using an ISS threshold of 15 [12,13,14,15]. Simple imaging was defined as x-rays and ultrasound, advanced imaging as computed tomography (CT), whole-body CT (WBCT), and magnetic resonance imaging (MRI) within 4 h of arrival [16].

### 2.4. Bias

To avoid bias and to reflect the entire spectrum of major trauma patients (with and without TTA), we used a multilayered approach to data extraction from multiple sources, as mentioned above.

### 2.5. Statistics

Record-based information was entered in a Microsoft Excel spreadsheet (Microsoft, Redmond, WA, USA). Further patient details were extracted from the EMR. After completion, the data were imported into IBM Statistical Software Package for Macintosh (SPSS, version 26), and the dataset was anonymized. Data were analyzed in SPSS. Analyses were performed with 2-sided tests and *p* < 0.05 was considered significant for all tests. Groups were compared using Fisher’s exact tests for categorical variables (due to low expected cell values); due to mostly non-normal distribution, associations between variables were analyzed using Spearman correlations. Correlation coefficients can be interpreted as follows: values between 0.3 and 0.5 indicate a low linear association, between 0.5 and 0.7 a moderate linear association, and between 0.7 and 0.9 a high linear association. To investigate the probability of having to activate the trauma team, a logistic regression analysis was performed with the following predictors that were selected based on preliminary analysis: medicalized transport (no/yes), child age, child gender, and ISS (<15 vs. ≥15).

## 3. Results

**Figure 1:**
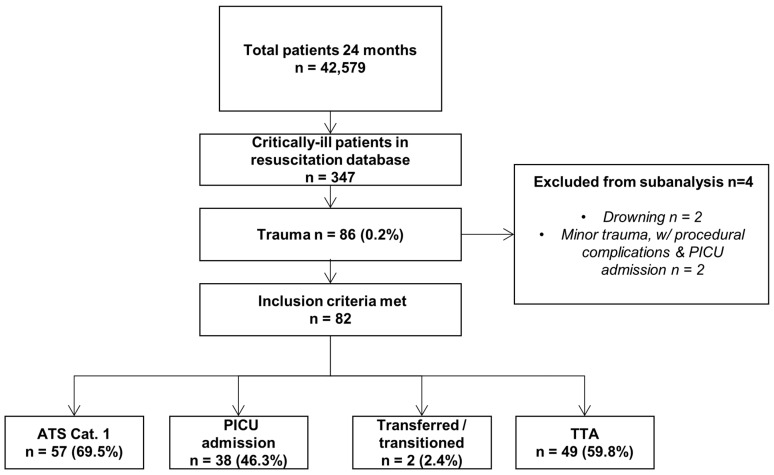
Patient enrolment.

Over the 2-year period, the PED recorded a total of 42,579 visits. A total of 347 patients, 0.8% of all visits, were identified in the resuscitation database. Of these, 86 were patients with a trauma diagnosis; after 4 patients were excluded, 82 matched one or more of the study inclusion criteria (Figure 1).

### 3.1. Patient Characteristics and Mechanism of Injury

Patient characteristics are shown in Table 1. The cohort comprised 73.2% males, and the median age was 8 years (IQR: 4.8–13.0 y). The median ISS overall was 9 (IQR 4–14), the major trauma group (19/23.2%) had a median ISS of 22 (IQR 17–26) (Table 2).

ATS category 1 was recorded in 69.5% of the cohort, and 46.3% required PICU admission.

Falls were the most frequent mechanism of injury (MOI), both in the major trauma group and overall. Road-traffic-related injuries had the highest relative risk of major trauma (Table 1 and Table 3).

Relationships between MOI and injury severity are shown in Table 1. There were no cases of non-accidental trauma meeting any of the inclusion criteria. There was no statistically significant difference in MOI across gender (χ^2^ (4,N = 82) = 2.335, *p* = 0.679).

### 3.2. Mode and Time of Arrival

Most patients arrived via medicalized transport (ambulance 52.4%, helicopter emergency service [HEMS] 31.7%), but 11 patients (13.4%) presented as walk-ins; of these, 4 (4.9% of all patients) received a TTA. More trauma patients presented on the weekends than during the week, but there was no statistical difference in TTAs (Table 2). TTAs were most common in morning shifts and rare during night shifts (n = 3). There was no significant association between trauma severity and shift of presentation (*p* = 0.872). However, there was an increase in trauma patients during warmer months (63.4% from April to September; Table 1).

### 3.3. Trauma Team Activation and Triage

Forty-nine patients (59.8%) in the cohort received a TTA. Of these, 39 patients (47.6%) were ATS 1, and 12 (14.6%) were major trauma. Of the 33 trauma patients without a TTA (40.2%), 18 (22.0%) were assigned ATS 1, and only 7 (8.5%) were major trauma. There were no differences across injury severity and TTA, although higher acuity triage categories (ATS 1 or 2) were significantly associated with TTA (*p* = 0.013; Table 2).

Multivariate logistic regression analysis showed that patients arriving via medicalized transport were more likely to receive a TTA (OR 4.4; 95%CI 1.2, 15.8). There were no associations between TTA and age (OR 1.0 [95%CI 0.9, 1.1]), gender (OR 0.8 [95%CI 0.2, 2.3]), or ISS (OR 1.3 [95%CI 0.5, 4.2]). Lifesaving interventions were rare (Supplementary Table S1—Lifesaving Interventions).

### 3.4. Imaging

Most patients in the study cohort (76 patients; 92.7%), received imaging. Of these, 48 patients (63.2%) had advanced imaging. Six patients with burns, contusions, or minor penetrating injury required no imaging. Patients over 6 years of age accounted for 69.8% of all CTs performed. There was no significant difference between the use of focused CT or WBCT and MOI (*p* = 0.114) or age group (<6 y vs. >6 y) (*p* = 0.736). However, an association between the use of type of CT scan and injury severity can be noted (see Table 4).

### 3.5. ED Disposition and Outcomes

Eighty patients survived (97.6%). The PICU and the inpatient ward each received 38 patients (46.9%). Two patients died, one in the PED due to injuries to the chest and abdomen and another in the PICU due to severe head injury (defined as AIS severity 3 or higher). Three patients (3.7%) were discharged home, and two patients were transferred out. One patient was transferred directly to the national burns center after initial treatment in the OR. A 15-year-old patient was transferred to the adult spine service with a lumbar spine fracture directly after resuscitation in the trauma bay. A breakdown of disposition and injury severity is shown in Table 4. There was no statistically significant association between ATS triage and disposition (*p* = 0.399), or between ATS triage and the need for PICU admission (*p* = 0.079). Admission to the inpatient ward was significantly associated with an ISS <15 (*p* < 0.001). Significant differences in length of stay (LOS) were seen between injury severity groups (*p* < 0.001). The majority LOS of ISS < 15 was less than 3 days (n = 33, 40.2%). The Spearman correlation coefficient was computed to assess for ISS and hospital LOS, which showed a significant, though moderate correlation between the variables (r = 0.577; *p* ≤ 0.001). This is also reflected in the median LOS of 7 days (IQR 4–9.5 y) for major trauma and 2 days (IQR 2–5 y) for minor trauma with ISS < 15.

### 3.6. Injury Severity

**Figure 2:**
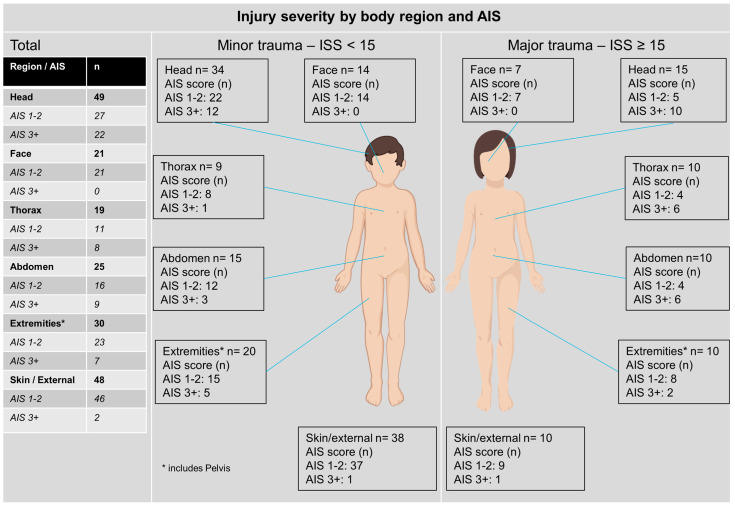
Injury severity overall, by body region and abbreviated injury score (AIS); by ISS (injury severity score) categories (for gender equality, body shapes of both sexes are shown).

There were no significant age differences between major and minor trauma groups (*p* = 0.46; Table 1 above). A higher proportion of males (25%, n = 15/60) had major trauma but this difference was not significant (*p* = 0.768; Table 1). The distribution of anatomical regions injured is depicted in Figure 2. In particular, injuries of AIS 3 severity or greater were most frequent in the head (12 patients), followed by the abdomen, chest, and extremities.

## 4. Discussion

### 4.1. Key Results

To address a gap in knowledge in pediatric trauma in Switzerland, we investigated a prospective cohort of major trauma patients. Actual or potential major trauma was rare, comprising only 0.2% of all presentations to the study PED in the study period. Pediatric trauma presentations in our geographical area were mostly male, arrived via medicalized transport, and consisted almost exclusively of blunt injuries, with falls being the most common MOI and road-traffic-related trauma the most severe. More than half of all patients received advanced imaging. Mortality was only 2.6% of the study cohort.

Svantner et al. analyzed 6 years of resuscitation registry data in a single center in western Switzerland [6]. The demographics were similar to our cohort. The median ISSs of Svantner et al.’s report and this study were both 9; in their report, 30% were major trauma (ISS > 15), and in our cohort, 23%. Major trauma in both cohorts had a median ISS of 22. The findings for MOI are also very similar.

Many other studies from high-income countries have examined major trauma (ISS > 15) at their centers, some covering over a decade. Our findings are in line with many other studies in the literature.

European and Australian studies mostly reported blunt injury mechanisms [3,17,18,19,20], with falls and traffic-related injuries most common [3,17,18,19,20,21,22]. MOI to preschool children differ widely among the studies, mostly because infants are not represented as a separate group. Nevertheless, severe burns are amongst the most common injury mechanisms in this age group [19,23]. Studies with an infant subgroup report non-accidental trauma as the most frequent injury in infants [19,20].

Mortality in other studies ranged from 3% to 14% [3,18,20,21,22], but inclusion criteria differ among studies. Deaths were often associated with road traffic [17,24] and head injury [17,23]. Many publications reported a median age around 7–8 years [3,17,20]. Moreover, a tendency was reported for arrival via HEMS to be associated with major trauma [18,21]. Our cohort showed no significant difference between HEMS and road ambulance in injury severity. Finally, penetrating injury and assaults seem to play a minor role in the pediatric population of high-income countries except for the United States [23]. However, this may depend partly on the age limit that defines the pediatric trauma population—whether 16, 18, or 21 years—in addition to the well-recognized higher levels of firearm injury in that country.

In our cohort, severe burns were observed only in patients below 6 years of age, which aligns with previous reports [23], but this may be influenced by the fact that older patients may be transferred directly to a national burns center [19]. In the period we analyzed, no patients under 6 years presented due to winter sports. Generally, sports and play injuries were more frequent above 6 years (see Table 3). Of eight traumas with ISS > 24, four were under 6 years, and all were male. Of the females in the cohort, only seven were under 6 years—two were ISS > 15, with one burn and one traumatic brain injury from road traffic. Some 21 males were under 6 years, comprising 75% of this young patient subgroup.

Both deaths were under 6 years and male. One was caused by a kids’ scooter colliding with a car, the other by a vehicle rolling over the chest and abdomen.

It has been suggested that an ISS > 12 based on 2008 AIS coding functions similarly to an ISS > 15 based on earlier AIS versions in identifying patients at higher risk of dying after injury [25]. In our cohort, an extra four patients would have qualified as major trauma if this alternative threshold had been used. Conversely, other authors have suggested using higher ISS thresholds in pediatrics due to lower mortality rates in pediatric trauma. However, this would result in far smaller patient numbers and potentially preclude useful evaluations of the quality of trauma care.

### 4.2. Trauma Team Activation

TTA was more frequent with medicalized transport, but there was no association with injury severity, and this finding did not change with transport by air or road. Trauma patient numbers could vary at similarly sized centers lacking Level 1 status. Criteria for TTA are well known to differ amongst institutions [26], leading to significant variations in the frequency of trauma calls due to the adoption of different activation thresholds. These activation criteria lack evidence [26]. TTA criteria must balance overtriage, which can strain resources, with undertriage, and potentially worse patient outcomes [27]. Calculated preclinical scores are imperfect [28], and the poor concordance between TTA and final injury severity leaves room for improvement [29]. At our center, TTA was a poor predictor of major trauma status as defined by ISS > 15, with a specificity of 41% (Table 2). In part, this may have been due to the study selection criteria, which included TTA patients, but may also have reflected poor performance of the TTA criteria used. The poor performance is likely driven by two TTA triggers: MOI and prehospital TTA request. Intervention-based measures have been found to perform better than the well-established major trauma definition [30,31], including as a means of evaluating TTA criteria [32]. Centers should review TTA criteria regularly to ensure that they identify patients who will benefit from a trauma team [33,34].

### 4.3. Imaging

Advanced imaging was used for decision making in 58% of all patients in the cohort. There was a significant association between injury severity and CT scanning, although 23 patients with an ISS < 10 received CT imaging. The reasons for ordering a CT scan or WBCT are hard to elucidate in retrospect. The reported MOI may play a role, although it has been suggested that MOI should not be the sole indication to image [35]. Other studies have reported a low adherence to imaging guidelines [36,37], raised concerns about the use of CT in hemodynamically stable patients [38], and shown high variability in imaging practice among trauma centers [39,40]. For patients in our cohort, a low-dose WBCT protocol was in place, limiting radiation exposure below the annual background radiation dose [41]. Thus, imaging should take place at a pediatric trauma center whenever possible [37,39,40]. Quality improvement initiatives elsewhere have demonstrated that CT imaging can be reduced without compromising care [42].

### 4.4. Limitations

Being a retrospective analysis of prospectively collected data, this study has inherent limitations. The data are derived from a single center located in a high-income country, making it challenging to generalize the findings. Moreover, it remains uncertain whether the results can be considered nationally representative. However, since our area of service is geographically confined, which minimizes the risk of patients seeking care elsewhere or bypassing our institution, this may mitigate the influence of referral bias. ISS was not used as a parameter for the identification of patients, because this is a calculated score. While there are several trauma scores with physiological parameters, we only used the anatomy-based ISS, as it is the most frequently used trauma score. In the absence of a comprehensive trauma registry and a comprehensive EMR, patients not fulfilling identified inclusion criteria could have been missed from the pyramid of injury [43]. However, one of the study’s strengths is its prospective data collection from multiple sources. Through this approach, patients who could have been missed in one data source were identified using another.

### 4.5. Outlook, Measures, and Prevention

The leading major trauma mechanisms in our cohort were falls and road-traffic-related injuries. Both of these MOI are amenable to injury prevention initiatives, and trauma prevention campaigns could help to avoid injuries [44]. Unfortunately, there is no national trauma register that includes data on severely injured children; some centers have their own registries [6], and government bodies provide non-specific epidemiologic data [44]. A comprehensive national trauma register could help to increase the efficacy and quality of pediatric trauma care. Despite an initiative by the national pediatric surgery society to introduce such a register in 2011 [7], this project has not transpired. A PEM-driven solution could be a minimal trauma dataset, similar to the neonatal minimal dataset established by the Swiss Society of Neonatology [45]. In our cohort, TTA did not correlate with injury severity, and balance between over- and undertriage is a continuing challenge at the prehospital and ED interface. Educators should bear in mind that because major trauma is a rare scenario, even in busy PEDs and dedicated pediatric trauma centers, specific training and simulation programs should have high priority [46].

## 5. Conclusions

Only a small percentage of patients in the PED arrive with major trauma, and trauma team activation correlates poorly with injury severity. This lack of exposure to severely injured patients emphasizes the value of simulation and deliberate learning activities for all PED professionals. Our research suggests that maintaining the PED’s capacity to manage severely injured children should be given high priority. In many cases in our cohort, severe injury mechanisms were avoidable, which should provide an impetus for additional efforts toward injury prevention.

## Figures and Tables

**Table 1:** Demographics, injury event, and mechanism of injury.

	Minor (ISS < 15)	Major (ISS ≥ 15)	Total n (%)	Significance Testing
*Sex*				*p* = 0.768
**Male**	45 (54.9)	15 (18.3)	60 (73.2)	
**Female**	18 (22.0)	4 (4.9)	22 (26.8)	
**Total**	63 (76.8)	19 (21.2)	82(100)	
*Age*				*p* = 0.631
**0–3**	10 (12.2)	4 (4.8)	14 (17.1)	
**4–7**	17 (20.7)	7 (8.5)	24 (29.3)	
**8–11**	14 (17.1)	2 (2.4)	16 (19.5)	
**12–15**	22 (26.8)	6 (7.3)	28 (34.1)	
*Mechanism of injury*				*p* = 0.388
**Sports**	18 (22.0)	2 (2.4)	20 (24.4)	
**Road traffic related ^#^**	12 (14.6)	5 (6.1)	17 (20.7)	
Falls *	24 (29.3) *	8 (9.8)	32 (39.0)	
**Blunt trauma**	8 (9.8)	3 (3.7)	11 (13.4)	
**Burns**	1 (1.2)	1 (1.2)	2 (2.4)	
*Seasonality*				
**Q1 (Jan** **–** **Mar)**	11 (13.4)	3 (3.7)	14 (17.1)	
**Q2 (Apr** **–** **Jun)**	19 (23.2)	9 (11.0)	28 (34.1)	
**Q3 (Jul** **–** **Sep)**	21 (25.6)	3 (3.7)	24 (29.3)	
**Q4 (Oct** **–** **Dec)**	12 (14.6)	4 (4.9)	16 (19.5)	

Legend: ISS—Injury Severity Score, ^#^ includes motor vehicle accidents and motor vehicle vs. pedestrian, * includes one impalement of lower leg by a wood branch.

**Table 2:** Emergency department reception (trauma activation, transport, triage, and timing).

	TTA	No TTA	Total n (%)	Significance Testing
*Transport mode*				*p* = 0.027
**Walk-in**	4 (4.9)	7 (8.5)	11 (13.4)	
Primary care *	0	2 (2.4)	2 (2.4)	
**Ambulance**	25(30.5)	18 (22.0)	43 (52.4)	
**HEMS**	20 (24.4)	6 (7.3)	26 (31.7)	
	49 (59.8)	33 (40.2)	82 (100)	
*Triage category*				*p* = 0.013
**ATS1**	39 (47.6)	18 (22.0)	57 (69.5)	
**ATS2**	9 (11.0)	9 (11.0)	18 (22.0)	
**ATS 3&4**	1 (1.2)	6 (7.3)	7 (8.5)	
*Day of presentation*				*p* = 0.344
**Weekday**	35 (42.7)	20 (24.4)	55 (67.1)	
**Weekend**	14 (17.1)	13 (15.9)	27 (32.9)	
*Shift of presentation*				*p* = 0.872
**08:00 h** **–** **16:00 h**	27 (32.9)	16 (19.5)	43 (52.4)	
**16:00 h** **–** **23:00 h**	19 (23.2)	15 (18.3)	34 (31.5)	
**23:00 h** **–** **08:00 h**	3 (3.7)	2 (2.4)	5 (6.1)	
*Trauma severity*				*p* = 0.795
**Major (ISS ≥ 15)**	12 (14.6)	7 (8.5)	19 (23.2)	
**Minor (ISS < 15)**	37 (45.1)	26 (31.7)	63 (76.8)	

Legend: ISS—Injury Severity Score, TTA—trauma team activation, ATS—Australasian Triage Scale, HEMS—helicopter emergency service, * patients from rural areas may be referred by primary care providers and transported by caregivers.

**Table 3:** Breakdown of injury mechanisms in detail.

Mechanism of Injury	<6 Years n (%)	>6 Years n (%)	Total n (%)
*Falls*			
**Fall > 3 m**	7 (8.5)	15 (18.3)	22 (26.8)
**Fall < 3 m**	6 (7.3)	4 (4.9)	10 (12.2)
*Sports injury*			
**Sporting/play injury**	2 (2.4)	9 (11.0)	11 (13.4)
**Winter sports injury**	-	9 (11.0)	9 (11.0)
*Road traffic*			
**Pedestrian vs. MV**	3 (3.7)	6 (7.3)	9 (11.0)
**MVA**	2 (2)	6 (7.3)	8 (9.8)
**Blunt trauma, NS**	6 (7.3)	5 (6.1)	11 (13.4)
**Burns**	2 (2.4)	-	2 (2.4)
**Total**	28 (34.1)	54 (65.9)	82 (100)

Legend: NS, not further specified; MV, motor vehicle; MVA, motor vehicle accident.

**Table 4:** Emergency department management—imaging and disposition.

	Minor (ISS < 15), n = 63	Major (ISS ≥ 15), n = 19	Total n (%)	Significance Testing
*Imaging*				
**No imaging**	5 (6.1)	1 (1.2)	6 (7.3)	*p* = 0.014
**X-ray only**	5 (6.1)	1 (1.2)	6 (7.3)	
**Ultrasound**	11 (13.4)	1 (1.2)	12 (14.6)	
**CT focused**	21 (25.9)	5 (6.1)	26 (31.7)	
**WBCT**	7 (8.5)	10 (12.2)	17 (20.7)	
**MRI**	1 (1.2)	0	1 (1.2)	
**US & X-ray**	10 (12.2)	0	10 (12.2)	
**WBCT & MRI**	3 (3.7)	1 (1.2)	4 (4.9)	
*Imaging categories*				*p* = 0.008 (n = 76) *
Simple Imaging * **(XR, US)**	26 (34.2)	2 (2.6)	28 (36.8) *	
Advanced Imaging * (CT, WBCT, MRI)	32 (42.1)	16 (21.1)	48 (63.2) *	
*CT imaging analysis (n = 43)*				*p* = *0.011*
- ***CT focused***	*21 (48.8)*	*5 (11.6)*	*26 (60.5)*	
- ***WBCT only***	*7 (16.3)*	*10 (23.3)*	*17 (39.5)*	
*Disposition ^+^*				*p* < 0.001 *^+^*
**Outpatient**	3 (3.7)	0	3 (3.7)	
**Inpatient**	36 (44.4)	2 (2.5)	38 (46.9)	
**PICU**	22 (27.2)	16 (19.8)	38 (46.9)	
**Transfer**	2 (2.5)	0	2 (2.4)	
**Death in PED**	0	1 (1.2)	1 (1.2)	
**Total**	63 (77.8)	18 (22.2)	82 (100)	

Legend: CT—computed tomography, ISS—Injury Severity Score, MRI—magnetic resonance imaging, PICU—pediatric intensive care unit, TTA—trauma team activation, US—ultrasound, WBCT—whole body CT, XR—X-ray, ^+^ n = 81, * total n = 76.

## Data Availability

The datasets generated and/or analyzed during the current study are not publicly available as this is beyond the scope of the ethics approval but are available from the corresponding author on reasonable request.

## Supplementary Materials

The following supporting information can be downloaded at: https://www.mdpi.com/article/10.3390/children10081377/s1, Supplementary Table S1—Lifesaving Interventions in the trauma bay 2018–2019.
