# Supplementary material for: Pediatric Trauma and Trauma Team Activation in a Swiss Pediatric Emergency Department: An Observational Cohort Study
Source: Children (Basel). 2023 Aug 11;10(8):1377. doi: 10.3390/children10081377 (PMC10453385; doi:10.3390/children10081377)
Supplement: Supplementary file 1 [file children-10-01377-s001.zip › children-2502434-supplementary.pdf]

Supplementary Table S1. Lifesaving interventions in the trauma bay 2018-2019.

**Lifesaving interventions**	**n**
Advanced airway management	2 (both intubations)
Cardiopulmonary resuscitation	1
Central line placement	0
Chest drain insertion	2
Laparotomy and thoracotomy	1
Intraosseous line placement	1
Massive transfusion protocol activated	0
